# Changes in Empathy during Medical Education: An Example from Turkey

**DOI:** 10.12669/pjms.335.13074

**Published:** 2017

**Authors:** Fusun Artiran Igde, Mustafa Kursat Sahin

**Affiliations:** 1 Fusun Artiran Igde, Associate Professor, Ondokuz Mayis University, Faculty of Medicine, Department of Family Medicine, Samsun, Turkey; 2 Mustafa Kursat Sahin, Assistant Professor, Ondokuz Mayis University, Faculty of Medicine, Department of Family Medicine, Samsun, Turkey

**Keywords:** Empathy, Medical students, JSE, Turkey

## Abstract

**Objective:**

Empathy is a key element of patient– physician communication; it is relevant to and positively influences patients’ health. In this study we aimed to present the Turkey example for the empathy change during the medical faculty training.

**Methods:**

This cross-sectional study was carried out at Ondokuz Mayis University, Faculty of Medicine, Department of Family Medicine, Samsun, Turkey. in first three years students of medicine during September 2014 to June 2015. Turkish adapted form of the student version of Jefferson empathy scale and sociodemographic questionnaire was used and data was assessed with the SPSS program.

**Results:**

Total 511 students (52.5% female, 47.5% male), from first three years of medical faculty participated in the study. Two hundred thirty three (44.5%) students were from the First Year class, while 130 (27.1%) and 148 (28.4%) of them were from Second and Third Year respectively. The mean age was 20.63±2.73 years. Significant differences in the empathy scores were observed among first three years in medical school as like as between gender.

**Conclusion:**

Although medical schools tend to raise students with higher empathy levels, medical education itself is more scientific based than humanistic approach, and makes medical students more tough and insensitive to the problems of patients. Patient-centered approach and empathy training should be implemented in the curriculum during whole medical education.

## INTRODUCTION

Empathy has been defined as a model containing cognitive as well as emotional fields^1^ that involves the capability to understand another person’s inner experiences and feelings and an ability to understanding the external world from the other person’s viewpoint.^2^,^3^

In medical education several skills that contribute towards the health care delivery abilities are supposed to be strengthened by empathy and medical schools are increasingly aware of their role in the improvement of empathy of their students. However, there are concerns that student empathy may deteriorate during undergraduate medical education^4^ and this situation is considered strongly related with patient health and well-being.^5^

For this purpose, we examined the level of empathy change in first three years of undergraduate medical students in Turkey. According to the literature review, our first objective was to compare empathy levels in medical students in first three pre-clinical years in order to define whether the decline in empathy also occurs in Turkish students. We also assessed gender differences, specialty choices in student levels of empathy, and observed the relation with academic performance.

## METHODS

This study was conducted at Ondokuz Mayis University, Faculty of Medicine, Department of Family Medicine, Samsun, Turkey. in first three years students of medicine during September 2014 to June 2015. Students in first three years of medical faculty in academic year 2014-2015 were included. The participants were 511 of whom 233 were first year, 130 were second year and 148 were third year medical students. Two hundred forty three (47.5%) of them were males, and 268 (52.5%) were females. The mean age was 20.6±2.7 years. Ethical approval was obtained from Institutional Ethical Committee. A sociodemographic questionnaire including age, gender, socioeconomic factors, family support, friends, speciality choice was developed by the authors according to the literature for the study.

### Student Version of the Jefferson Scale of Physician Empathy

The Jefferson Physician Empathy Scale was devised as the result of a series of studies using US doctors and medical students.^6^ The scale includes 20 items (10 items positively worded and 10 items negatively worded) answered on a 7-point Likert scale from 1 (strongly disagree) to 7 (strongly agree). The score interval is 20-140, higher scores show higher empathic consistency. For use with a UK sample the substitution of the term “doctor” for the term “physician” used in the original scale items was considered appropriate. Example items are: “The best way to take care of a patient is to think like a patient”, “Emotion has no place in the treatment of medical illness” (reverse scored), “Patients feel better when their feelings are understood by their doctors”.^7^

Student Version of the Jefferson Empathy Scale was adapted to the Turkish population by Gonullu et al.^7^ The Cronbach-alpha for the entire scale was 0.83 and factor level with Cronbach’s alphas of 0.83, 0.70 and 0.60 for the “perspective taking”, “compassionate care” and “standing in the patient’s shoes” components respectively. The three-dimensional structure of the JSPE was confirmed by CFA except item 18.^7^

### Statistical analyses

Statistical analysis was evaluated using SPSS software version 22.0. (SPSS Inc, CA, USA). Results are expressed as mean and standard deviation (SD). Differences between groups were analyzed with the student t-test, Mann-Whitney U test, ANOVA or Kruskall Wallis for independent samples. Differences in categorical variables between the two groups were analyzed by the chi-square test. Correlation analyses were performed using the Pearson and Spearman correlation and regression analyses was performed when necessary. A value of p < 0.05 was considered statistically significant.

## RESULTS

A total of 511 medical students of whom 233 were first year, 130 were second year and 148 were third year were included. (Table-I). Reliability analyses were calculated by Cronbach’s alpha for 20 item Student Version of the Jefferson Scale of Physician Empathy (alpha = 0.88) and 0.70, 0.80, 0.87 for the “perspective taking”, “compassionate care” and “standing in the patient’s shoes” subscales respectively.

**Table-I:** Sociodemographic results for first, second and third years students.

	*Year 1*	*Year 2*	*Year 3*
Age, mean (SD)	19.52 (1.9)	21.22 (1.2)	21.72 (3.8)
*Gender*			
Female	120 (51.5)	70 (53.8)	78 (52.7)
Male	113 (48.5)	60 (46.2)	70 (47.3)
Total	233 (100)	130 (100)	148 (100)
*Family Support*			
Very Good	107 (45.9)	64 (49.2)	46 (31.1)
Good	93 (39.9)	54 (41.5)	89 (60.1)
Not sure	14 (6.0)	6 (4.6)	8 (5.4)
Poor	11 (4.7)	0	2 (1.4)
Very poor	8 (3.4)	6 (4.6)	3 (2.0)
Total	233 (100)	130 (100)	148 (100)
*Anxious most of the time*			
Strongly agree	19 (8.2)	5 (3.8)	8 (5.4)
Agree	55 (23.6)	41 (31.5)	34 (23.0)
Not sure	54 (23.2)	17 (13.1)	19 (12.8)
Disagree	79 (33.9)	46 (35.4)	72 (48.6)
Strongly disagree	26 (11.2)	21 (16.2)	15 (10.1)
Total	233 (100)	130 (100)	148 (100)
*Speciality Choice*			
Technology oriented	113 (48.5)	45 (34.6)	77 (52.0)
Human oriented	120 (51.5)	85 (65.4)	71 (48.0)
Total	233 (100)	130 (100)	148 (100)
*Final grade of year*			
Passed	163 (70.0)	126 (96.9)	141 (95.3)
Failed	70 (30.0)	4 (3.1)	7 (4.7)
Total	233 (100)	130 (100)	148 (100)

The correlation between total score of empathy and academic performance was (r= -0.118, p=0.07) for year one, (r= -0.043, p=0.62) for year two, (r= -0.094, p=0.25) for year three. The means and standard deviations of the Student Version of the Jefferson Scale of Physician Empathy scores by gender and year groups are presented in Table-II. Significant gender differences were found for the whole sample on empathy and perspective taking, standing in the patient’s shoes of empathy subscales for year 1 and for the whole sample on empathy and compassionate care of empathy subscale for year three with females scoring higher than males.

**Table-II:** Mean (standard deviation) and p values of empathy scores for male and female students.

	*Year 1*			*Year 2*			*Year 3*		

	*Female*	*Male*	*p*	*Female*	*Male*	*p*	*Female*	*Male*	*p*
Empathy	76.94(10.23)	73.66(12.77)	0.03	58.86(4.60)	59.48(4.84)	0.45	59.87(3.52)	58.59(3.29)	0.02
Perspective	40.20(5.58)	38.19(6.64)	0.01	30.01(2.29)	30.47(3.02)	0.32	30.53(2.31)	30.41(2.40)	0.77
Compassionate	28.24(3.88)	27.44(5.10)	0.18	24.15(2.52)	24.31(2.69)	0.72	24.54(1.98)	23.38(2.55)	0.00
Patient’s shoes	8.50(1.25)	8.02(1.75)	0.01	4.69(1.64)	4.69(1.71)	0.99	4.79(1.31)	4.78(1.38)	0.96

(Perspective: Perspective Taking; Compssionate: Compassionate Care; Patient’s shoes: Standing in the Patient’s Shoes).

Regarding specialty choice within gender, female students preferred people-oriented specialties more than men (60% vs 47.5%, p=0.01).

### Comparison of Physician Empathy scores within year groups

**Fig.1:**
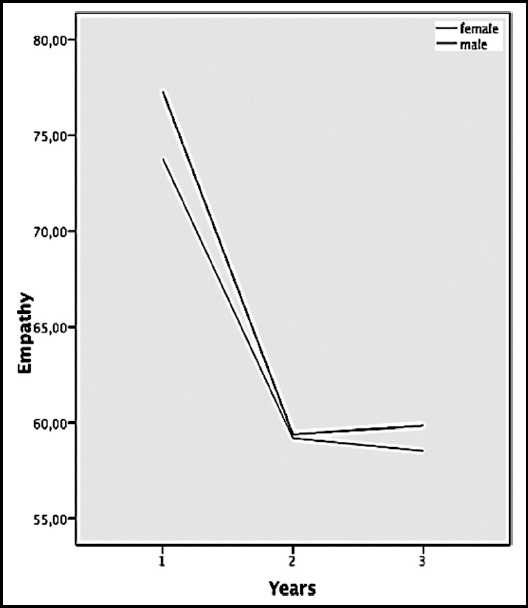
Empathy scores changes in years by sex.

According to ANOVA test there was a significant difference between year 1, 2 and 3 in Empathy (F=231.94, p=0.00) total scale and whole subscales (Table-III). Total empathy results according to gender and years are illustrated in Fig.1 which shows sharp decreasing empathy levels in both gender between Years 1 and 2. Post-hoc assessment indicated that the difference between male and female levels of empathy was significant in Year 1 (t=2.26, p=0.02) and Year 3(t=2.14, p=0.03).

**Table-III:** Comparison of Physician Empathy scores within year groups.

	ANOVA				

	*Sum of Squares*	*df*	*Mean Square*	*F*	*Sig.*
Perspective taking	9943.820	2	4971.910	238.698	.000
Compassionate care	1755.928	2	877.964	69.158	.000
Patient shoes	1573.459	2	786.729	342.042	.000
Empathy total score	32839.594	2	16419.797	231.941	.000

### Logistic Regression

Physician empathy scores significantly contributed to the family support of the students with a odds ratio (OR) 1.71, also as the years in faculty had increased, empathy scores significantly decreased with a OR -0.596.Table IV.

**Table-IV:** Logistic regression model for total empathy score with years, gender, family support, anxious most of the time, speciality choice.

		*Variables in the Equation*					

		*B*	*S.E.*	*Wald*	*df*	*Sig.*	*Exp(B)*
Step 1^a^	Years	-20.160	1.621	-.695	1	.000	-.596
	Family support	.541	.169	10.259	1	.001	1.717
	Being anxious	-.214	.125	2.913	1	.088	.807
	Speciality choice	.435	.286	2.319	1	.128	1.545
	Constant	17.571	1.610	.000	1	.991	4.702

a Variable(s) entered on step 1: years, gender, family support, anxious, speciality choice.

Although not statistically significant according to the regression analysis, students who are anxious had lower empathy score than less anxious [β=-.21, 95%CI (-.078,.003)]. People oriented speciality choosen students have marked higher [β=.43, 95%CI (-.005, 0.178)] empathy score than technology oriented speciality choosen students (Table-IV).

## DISCUSSION

Empathy is perceived as one of the individual abilities that defines efficiency in medicine and as an essential condition for “patient centered” care.^8^,^9^ Numerous authors have studied that the decline in empathy is a result of a self- protection that diminishes the pain and misery faced through clinical relations with patients and their families.^10^,^11^

In this study it was also noted a decrease in empathy level within years. Although mostly researchers mentioned that the third year is typically when students often deal with emotionally challenging and difficult situations while rotating through various clinical clerkship, their empathy declines as a protective defense mechanism^12^ we found a sharp decrease in empathy level after the first year of education in preclinical settings. On the other hand the mean score of our medical students empathy level was also quite low according to other studies.^13^,^14^ It may be thought that the social anxiety present in Turkey in recent years may cause this situation, but this cannot explain the significant decrease between first and the following years. In our faculty and other medical faculties in the country, there is no constructed empathy training in undergraduate medical education. Patient-physician communication and patient-centered approach are given as a skill training for the first three years in our school. While this training is about three hours per student in the second and third years, the time allocated to the patient-physician communication in the first year covers approximately 12 hours for each student. In addition, students in the first year also have a two-hour empathy skill training for each student who is experiencing three different disability situations (blind patient, hemiplegic patient using a wheelchair and foot-arm disabled patient using crutch), this could be the reason for the significantly higher levels of empathy in first year students.

Many studies have detected a relationship between speciality choice and empathy which found that students are likely to choose people-oriented specialties as family medicine, internal medicine, psychiatry, pediatrics had higher empathy scores than technology-oriented specialties, when controlled for gender effects.^15^-^17^ Although it’s not statically significant we also found that preferring people-oriented specialties as a career have higher empathy than students preferring technology-oriented specialties. As well as female students preferred people-oriented specialties more than men consisting with other authors.^10^,^18^

Previous findings on male/female differences in empathy^6^,^19^ were also somewhat confirmed, with higher scores being observed for females on all scales. Similarly our results shows that female medical students’ empathy levels were significantly higher than male for the first year of faculty. Although this difference disappear in second year, girl’s empathy level varied again in third year consisting evidence-based findings reported in the literature, attributing this difference to evolutionary and social learning factors.^2^,^20^-^24^

In the study, overall academic performance was found not to be associated with empathy in any of the year groups as like as many of studies.^25^

Although empathy is a critical issue in promoting patient-centered care, medical school curriculum force to shift the students toward scientific approach more than humanitarian attitude so students lose their empathy as they are subjected to challenges of practicing modern day medicine. Thus, development the learning environment for students to improve their empathy like increasing physician-patient communication, patient-centered clinical experience, standardized patient education during medical education is extremely important.

### Authors’ Contribution

**FAI and MKS** conceived, designed and did statistical analysis & editing of manuscript.

## References

[1] Rahimi-Madiseh M, Tavakol M, Dennick R, Nasiri J (2010). Empathy in Iranian medical students:A preliminary psychometric analysis and differences by gender and year of medical school. Med Teach.

[2] Hojat M, Gonnella JS, Nasca TJ, Mangione S, Veloksi JJ, Magee M (2002). The Jefferson Scale of Physician Empathy:further psychometric data and differences by gender and specialty at item level. Acad Med.

[3] Dehning S, Girma E, Gasperi S, Meyer S, Tesfaye M, Siebeck M (2012). Comparative cross-sectional study of empathy among first year and final year medical students in Jimma University, Ethiopia:steady state of the heart and opening of the eyes. BMC Med Educ.

[4] Hojat M, Mangione S, Nasca TJ, Rattner S, Erdmann JB, Gonnella JS (2004). An empirical study of decline in empathy in medical school. Med Educ.

[5] Little P, Everitt H, Williamson I, Warner G, Moore M, Gould C (2001). Preferences of patients for patient centred approach to consultation in primary care:observational study. BMJ.

[6] Hojat M, Mangione S, Nasca TJ, Cohen MJM, Gonnella JS, Erdmann JB (2001). The Jefferson Scale of Physician Empathy:development and preliminary psychometric data. Educ Psychol Meas.

[7] Gonullu I, Oztuna D (2012). Turkish Adaptation Of The Student Version Of Jefferson Scale Of Physician Empathy. Marmara Med J.

[8] Quince TA, Parker RA, Wood DF, Benson JA (2011). Stability of empathy among undergraduate medical students:a longitudinal study at one UK medical school. BMC Med Educ.

[9] Mead N, Bower P (2000). Patient-centredness:a conceptual framework and review of the empirical literature. Soc Sci Med.

[10] Chen D, Lew R, Hershman W, Orlander J (2007). A cross-sectional measurement of medical student empathy. J Gen Intern Med.

[11] Newton BW, Barber L, Clardy J, Cleveland E, O'Sullivan P (2008). Is there hardening of the heart during medical school?. Acad Med.

[12] Kay J (1990). Traumatic deidealization and the future of medicine. JAMA.

[13] Hojat M, Gonnella JS (2015). Eleven Years of Data on the Jefferson Scale of Empathy-Medical Student Version (JSE-S):Proxy Norm Data and Tentative Cutoff Scores. Med Princ Pract.

[14] Yucel H, Acar G (2016). Levels of empathy among undergraduate physiotherapy students:A cross-sectional study at two universities in Istanbul. Pak J Med Sci.

[15] Newton BW, Savidge MA, Barber L, Cleveland E, Clardy J, Beeman G (2000). Differences in medical students'empathy. Acad Med.

[16] Halpern J (2003). What is clinical empathy?. J Gen Intern Med.

[17] Roter DL, Stewart M, Putnam SM, Lipkin M, Stiles W, Inui TS (1997). Communication patterns of primary care physicians. JAMA.

[18] Avasarala SK, Whitehouse S, Drake SM (2015). Internship and Empathy:Variations Across Time and Specialties. South Med J.

[19] Hegazi I, Wilson I (2013). Maintaining empathy in medical school:it is possible. Med Teach.

[20] Hojat M, Gonnella JS, Nasca TJ, Mangione S, Vergare M, Magee M (2002). Physician empathy:definition, components, measurement, and relationship to gender and specialty. Am J Psychiatry.

[21] Hojat M, Mangione S, Nasca TJ, Gonnella JS, Magee M (2005). Empathy scores in medical school and ratings of empathic behavior in residency training 3 years later. J Soc Psychol.

[22] Sherman JJ, Cramer A (2005). Measurement of changes in empathy during dental school. J Dent Educ.

[23] Ward J, Schaal M, Sullivan J, Bowen ME, Erdmann JB, Hojat M (2009). Reliability and validity of the Jefferson Scale of Empathy in undergraduate nursing students. J Nurs Meas.

[24] Neumann M, Edelhauser F, Tauschel D, Fischer MR, Wirtz M, Woopen C (2011). Empathy decline and its reasons:a systematic review of studies with medical students and residents. Acad Med.

[25] Hojat M, Gonnella JS, Mangione S, Nasca TJ, Veloski JJ, Erdmann JB (2002). Empathy in medical students as related to academic performance, clinical competence and gender. Med Educ.

